# Presence and reactivities of antibodies directed to citrullinated peptides in a Swedish JIA cohort

**DOI:** 10.1186/s12969-025-01177-1

**Published:** 2025-11-22

**Authors:** Raya Saleh, Erik Sundberg, Monika Hansson, Linda Mathsson-Alm, Karl Skriner, Guy Serre, Karin Lundberg, Leonid Padyukov, Helena Erlandsson Harris

**Affiliations:** 1 Division of Rheumatology, Center for Molecular Medicine, Department of Medicine Solna, Karolinska Institutet, Karolinska University Hospital, Stockholm, Sweden; 2 Pediatric Rheumatology unit, Astrid Lindgren Children’s Hospital, Karolinska University Hospital, Stockholm, Sweden; 3 Department of Immunology, Genetics and Pathology, Uppsala University, Thermo Fisher Scientific Uppsala, Uppsala, Sweden; 4 Charité Universitätsmedizin Berlin, Humboldt University of Berlin, Berlin, Germany; 5 INFINITY, Inserm - CNRS - Université Toulouse III, Toulouse, France; 6 Broegelmann Research Laboratory, Department Clinical Medicine, University of Bergen, Bergen, Norway

**Keywords:** Juvenile idiopathic arthritis (JIA), Anti-CCP antibodies, Antibodies against citrullinated proteins/Peptides (ACPA), Filaggrin, Fibrinogen, Vimentin, Α-enolase, Heterogeneous nuclear ribonucleoprotein (hnRNP), HLA-DRB1 shared epitope

## Abstract

**Background:**

Juvenile idiopathic arthritis is a heterogeneous group of chronic inflammatory joint diseases in children. The presence and role of antibodies targeting citrullinated proteins remains unclear in this patient group. This study aimed to assess antibodies against citrullinated peptides in a Swedish cohort of patients with juvenile idiopathic arthritis and to explore their associations with clinical features and genetic markers.

**Methods:**

Plasma samples from 334 patients with juvenile idiopathic arthritis were analyzed for antibodies against cyclic citrullinated peptides using enzyme-linked immunosorbent assay. A subset of 100 patients underwent detailed profiling of antibody reactivity to 15 citrullinated peptides and their non-citrullinated counterparts using a multiplex microarray. Rheumatoid factor, antinuclear antibodies, and HLA-DRB1 genotypes were also assessed.

**Results:**

Antibodies against cyclic citrullinated peptides were detected in 6.2% of patients, with the highest prevalence in the rheumatoid factor–positive polyarthritis subtype. These patients were older at disease onset and more frequently carried HLA-DRB1 shared epitope alleles. Among the 100 profiled patients, 85% of those positive for cyclic citrullinated peptide antibodies showed reactivity to at least one citrullinated peptide, compared to 19% of antibody-negative patients. The most frequent reactivities were directed against peptides derived from filaggrin, fibrinogen, vimentin, and enolase. Reactivity to non-citrullinated peptides was minimal. A significant association was found between reactivity to citrullinated vimentin and the presence of HLA-DRB1 shared epitope alleles.

**Conclusions:**

This study reveals a distinct pattern of antibody reactivity to citrullinated peptides in a subset of patients with juvenile idiopathic arthritis, suggesting a biologically distinct subgroup with similarities to adult rheumatoid arthritis. These findings identify ACPA-positive JIA as a unique subgroup of young patients and underscore an opportunity to investigate early molecular events driving the immune response to citrullinated proteins and the pathogenesis of arthritis.

**Supplementary Information:**

The online version contains supplementary material available at 10.1186/s12969-025-01177-1.

## Background

Juvenile idiopathic arthritis (JIA) is a heterogeneous group of arthritis subtypes with a clinical onset before the age of 16, persisting for at least 6 weeks and having an unknown cause [1]. JIA is characterized by synovitis, which may lead to irreversible joint destruction, causing pain and functional disability. Thus, accurate diagnosis and intervention at an early stage of the disease are important for these patients. According to the International League of Associations for Rheumatology (ILAR) classification criteria, JIA is divided into seven mutually exclusive subtypes: oligoarthritis and polyarthritis, which are further divided into rheumatoid factor (RF)-positive and -negative polyarthritis, psoriatic arthritis, enthesitis-related arthritis (ERA), systemic JIA, and undifferentiated arthritis for cases that fit no category or fit more than one category [1].

No specific serological biomarker is defined for JIA, and diagnosis mainly depends on clinical manifestations. However, RF and antinuclear antibodies (ANAs) are routinely used for classifying and evaluating the clinical status of JIA. RF is found in 2–12% of JIA patients and is known as a predictor of physical disability, erosions, and failure to achieve remission [2, 3].

In recent years, patients have also been tested for the presence of anti-citrullinated protein/peptide antibodies (ACPAs) [2], commonly via commercial ELISAs based on synthetic cyclic citrullinated peptides (CCPs), such as the second-generation CCP2 test. Anti-CCP antibodies are highly specific (i.e., 98%) for rheumatoid arthritis (RA) and have a sensitivity of approximately 70% [4, 5]. The presence of anti-CCP antibodies is associated with HLA-DRB1 shared epitope (SE) alleles, and there is a gene‒environment interaction between SE and smoking in the development of ACPA-positive RA [6]. ACPAs can be detected several years before clinical manifestations of RA [7], and there is a heightened likelihood of transitioning to inflammatory arthritis when ACPAs are present [8]. Interestingly, both bone loss and lung disease have been described in ACPA-positive individuals without clinical arthritis [9, 10]. Recent evidence suggests that ACPAs are involved in the initiation rather than the progression of RA [11, 12]. Nevertheless, anti-CCP antibodies are currently used as diagnostic and prognostic markers in RA [5, 13] since they are also associated with disease severity, particularly erosive disease, in both RA and JIA [3, 14, 15].

The prevalence of anti-CCP antibodies in patients with JIA has varied across different studies. However, it has been more frequently recorded in the RF-positive polyarthritis subtype, which is also associated with HLA-DRB1 SE alleles [3, 16].

The anti-CCP test is designed to have high sensitivity and specificity for RA, but the exact nature of the antigen(s) used in the test and their biological relevance and pathological importance are not known [17, 18]. In RA patients, the ACPA response is known to be heterogeneous, different RA patients display reactivities against different citrullinated peptides and proteins, and there is large interindividual variability in ACPA reactivities [19–21]. Prior investigations of ACPA reactivities in JIA patients have described individual antibody responses to epitopes on citrullinated vimentin, collagen type II, fibrinogen and α-enolase, as detected by ELISA [22–26].

This study aimed to investigate the prevalence of anti-CCP antibodies in a well-characterized Swedish JIA patient cohort and to define the ACPA reactivity profile with a multiplex microarray platform capable of simultaneously detecting multiple citrulline reactivities. Additionally, as it remains to be established whether subclassification based on ACPA reactivity is clinically meaningful, we investigated the relationships of anti-CCP antibodies with patient characteristics and clinical parameters, HLA-DRB1 SE, RF and ANA.

## Methods

### Study population

Plasma samples were obtained from 334 JIA patients with active disease at Astrid Lindgren Children’s Hospital in Stockholm, Sweden, as part of the JIA sample collection (JABBA). Patients were enrolled and diagnosed according to the International League of Associations for Rheumatology (ILAR) criteria (Table 1) [1]. Plasma samples from 190 age- and sex-matched, joint-healthy children were included as references (mean age = 10.6 years; median = 12 years; age range: 4–17 years for healthy references, 1–18 years for JIA; 70% female). The reference samples were originally collected as part of “Barnens hälsoundersökning,” a population-based cohort of healthy children from the Stockholm region, which is also the region where JIA patients were recruited. The plasma samples were collected in EDTA tubes and stored at -80 °C until analysis. For JIA patients, patient- and disease-related data were extracted from the Swedish Juvenile Rheumatology Quality Registry and from medical records. The study was approved by the Regional Ethical Review Board in Stockholm (ethical permit numbers: 2009/1139-31 & 2010/165 − 32, 02-509 & Dnr 03–067), and all study participants/legal guardians provided written informed consent.

### Anti-CCP ELISA

Anti-CCP IgG antibodies were detected with Immunoscan CCPlus^®^ CCP2 ELISA (Euro-Diagnostica AB, Malmö, Sweden) according to the manufacturer’s instructions. The cut-off for positivity was set at 25 AU/mL according to the protocol for the analysis of RA samples. For each JIA patient, the earliest obtained plasma sample after diagnosis was analysed. Plasma samples from the reference cohort (*n* = 66) were analysed in parallel.

### Multiplex antigen array

An investigation of the fine specificity of the IgG antibody ACPA for citrullinated peptides and their arginine-containing counterparts was performed with a custom-made multiplex solid-phase microarray platform (Thermo Fisher Scientific, Uppsala, Sweden) as previously described [27]. The multiplex array included 15 citrullinated peptides derived from filaggrin (Fil_307-324_/cfc1-cyc), fibrinogen (Fibα_36-50_, Fibα_563-583_, Fibα_580-600_, Fibα_621-635_, Fibβ_36-52_, Fibβ_60-74_) [28], vimentin (Vim_2-17_, Vim_60-75_), α-enolase (CEP-1) and heterogeneous nuclear ribonuclear protein (hnRNP) (Pept Z1, Pept Z2, Pept 1, Pept 5, Pept Bla26) (details in supplementary Table 1). Reactivity against noncitrullinated arginine-containing peptides was investigated in parallel. The recorded fluorescence intensities were normalized and expressed as arbitrary units (AUs). The 98th percentile reactivity among 190 healthy references was used to determine cut-off values for each antigen.

### ANA and RF analysis

ANA and RF were measured by the certified clinical laboratory at Karolinska University Hospital as part of the clinical diagnosis, and information regarding seropositivity for each antibody was retrieved from the medical records. IgM RF was measured via the FEIA method (EliA^TM,^ Phadia AB, Uppsala, Sweden); the cut-off corresponds to 5% positivity in healthy adult blood donors and follows the ACR/EULAR guidelines for RA. ANA was measured by indirect immunofluorescence (IIF) (Hep-2 cells) with a cut-off set at a titre of ≥ 1:320 serum dilution, which corresponds to 3–5% positivity in healthy adult blood donors (in accordance with the Swedish laboratory guidelines for ANA).

### HLA-DRB1 genotyping and classification

JIA patients were genotyped via the Illumina assay InfiniumOmniExpressExome-8v1-4_A (Illumina Inc.) as part of a genome-wide association study. HLA-DRB1 genotypes were imputed using SNP2HLA (V.1.0) (http://www.broadinstitute.org/mpg/snp2hla/), and the dataset collected by the type 1 diabetes genetics consortium was used as the reference panel (*n* = 5225) [29]. The following HLA-DRB1 alleles were regarded as SE alleles: HLA-DRB1*01:01, *01:02, *01:05, *04:01, *04:04, *04:05, *04:08, *04:09, *04:10, *04:13, *10:01, *14:02, *14:06.

### Statistical analysis

Nonparametric statistics were used throughout the study. Differences in antibody levels were analysed via the Mann‒Whitney U test for independent groups. For comparison of the anti-CCP–positive and anti-CCP–negative groups, Fisher’s exact tests were used when appropriate, except for testing for age and disease duration, for which the Mann‒Whitney U test was used. Correlations of ACPA reactivities with HLA-DRB1 SE alleles were calculated with Fisher’s exact tests. The co-occurrence of ACPA fine specificities was assessed with Spearman correlation. The heatmap was generated using Python. P values were adjusted for multiple comparisons with the Benjamini‒Hochberg (BH) procedure, which controls the false discovery rate (FDR).

## Results

### Anti-CCP antibody levels

**Fig. 1:**
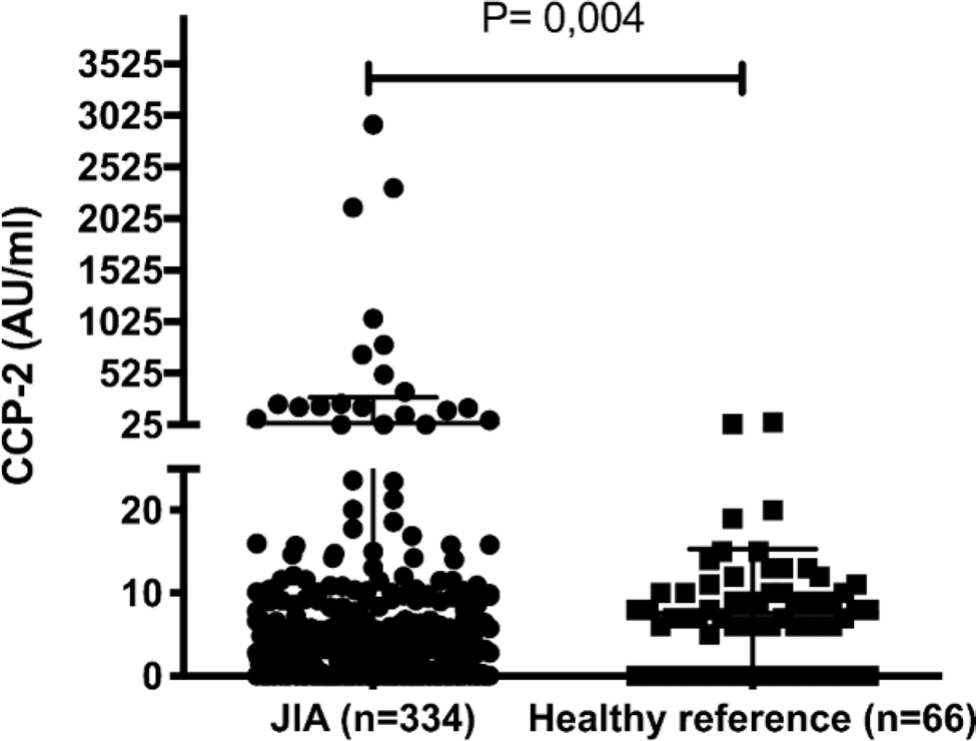
Anti-CCP antibody levels in JIA patients compared to healthy references. Anti-CCP levels were measured by ELISA for 334 JIA patients and 66 healthy references. Statistics calculated with Mann-Whitney test

Among the 334 JIA patients and 66 healthy references 21 JIA patients (6.2%) and two reference patients (3%) were positive for anti-CCP antibodies. Compared with the references, the JIA patients had significantly greater levels of anti-CCP (*p* = 0.004) (Fig. 1). Anti-CCP antibodies were detected in nine out of 13 (69%) RF-positive polyarthritis patients, whereas in the RF-negative polyarthritis subtype, only four out of 84 patients (4.8%) were positive. Among the patients with the oligoarthritis subtype, six out of the 170 patients (3.5%) were anti-CCP–positive, and among the patients with undifferentiated arthritis, two out of the four patients (50%) were positive. In the remaining subtypes, no anti-CCP reactivity was recorded. The characteristics and comparisons of the anti-CCP–positive and anti-CCP–negative JIA patients are presented in Table 1. Patients in the anti-CCP–positive subset were significantly older at disease onset, with a median age of 131 months (~ 11 years) *versus* 94 months (~ 8 years) in the anti-CCP–negative subset (*p* = 0.05), and they were also more frequently positive for RF (61.9% *versus* 1.6%, *p* < 0.04). There was no significant difference between the anti-CCP–positive and anti-CCP–negative groups in terms of ANA. RF-positive polyarthritis was more common in anti-CCP–positive patients (42.9% *versus* 1.3%, *p* < 0.04), as was undifferentiated arthritis (10% among anti-CCP–positive patients *versus* 0.6% among anti-CCP–negative patients, *p* = 0.05). No statistically significant difference for the other disease subtypes was observed. 62% of the anti-CCP–positive JIA patients had at least one HLA-DRB1 SE allele, whereas 33% of the anti-CCP–negative JIA patients did (*p* = 0.04). Disease duration did not differ significantly between the two groups (Table 1).

**Table 1:** Descriptive characteristics of Anti-CCP–positive and anti-CCP–negative JIA patients

Characteristics	Anti-CCP+ (*n* = 21)	Anti-CCP- (*n* = 313)	*P*-value	Adjusted *P*-value (FDR)
HLA-DRB1 SE+	13/21 (61.9%)	102/313 (32.6%)	< 0.01	0.04
Sex, Female	18/21 (85.7%)	216/313 (69.0%)	0.14	0.23
Age at onset, median months (Range)	131 (21–186)	94 (9–200)	0.01	0.05
Disease duration, median months (Range)	21 (2–94)	32 (0–185)	0.14	0.29
RF-positive	13/21 (61.9%)	5/313 (1.6%)	< 0.01	0.04
ANA-positive	10/21 (47.6%)	165/313 (52.7%)	0.66	0.66
Medication, Yes*	18/21 (85.7%)	191/313 (61.0%)	0.03	0.06
ACPA reactivity-positive	17/20 (85.0%)	15/80 (18.7%)	< 0.01	< 0.01
Polyarthritis RF-positive, *n* = 13	9/21 (42.9%)	4/313 (1.3%)	< 0.01	0.04
Polyarthritis RF-negative, *n* = 84	4/21 (19.0%)	80/313 (25.6%)	0.61	0.66
Oligoarthritis, *n* = 170	6/21 (28.6%)	164/313 (52.4%)	0.04	0.07
Entesitis related, *n* = 21	0/21 (0.0%)	21/313 (6.7%)	0.38	0.47
Psoriatic, *n* = 20	0/20 (0.0%)	20/313 (6.4%)	0.62	0.66
Systemic, *n* = 22	0/22 (0.0%)	22/313 (7.0%)	0.38	0.47
Undifferentiated, *n* = 4	2/20 (10.0%)	2/313 (0.6%)	0.02	0.05

ANA data was available for 332 patients, RF data was available for 218 patients and HLA allele frequencies were available for 300 patients. ACPA profiling was performed in 100 JIA patients (20 anti-CCP–positive, 80 anti-CCP–negative). * Of the CCP + patients, 3 had NSAIDs only, 4 had DMARDs only, 10 had bDMARDs, 1 predisolone and none tDMARDs. Of the CCP- patients 27 had NSAIDs only, 64 had DMARDs only, 79 had bDMARDs, 21 predisolone and none tDMARDs

### ACPA reactivities

To explore the profile of citrullinated peptide reactivities within our JIA cohort, we conducted an analysis using samples from 20 anti-CCP–positive and 80 anti-CCP–negative patients (100 patients in total for whom samples were available). The 80 anti-CCP–negative patients were selected to match the anti-CCP–positive patients in terms of age and sex.

A majority of anti-CCP–positive patients (85%) had reactivity to at least one citrullinated peptide, whereas 19% of anti-CCP–negative patients had reactivity (*p* < 0.01) (Table 1).

ACPA reactivity was significantly greater in the anti-CCP–positive group than in the anti-CCP–negative group across all investigated citrullinated peptides. The most prevalent responses in the anti-CCP–positive group were observed against Cit-Fil_307 − 324_ (60.0%), Cit-Fibα_36−50_ (50.0%), Cit-Fibβ_60−74_ (60.0%), Cit-Vim_60 − 75_ (60.0%), CEP-1 (55.0%), and Cit-Pept 5 (55.0%) (Table 2).

Notably, although the prevalence of all individual ACPAs in the anti-CCP–negative group was significantly lower than that in the anti-CCP–positive group, ACPA reactivities were still detectable in 18.7% of the anti-CCP–negative patients, with frequencies of up to 5% against CEP-1 and 6.2% against Vim60–75 (Table 2).

In contrast, reactivity to the corresponding arginine peptides was low or absent in both groups, with no statistically significant difference between the anti-CCP–positive and anti-CCP–negative groups for most peptides.

**Table 2:** ACPA reactivities in Anti-CCP–positive and anti-CCP–negative JIA patients

ACPA Reactivity	All JIA (*n* = 100)	Anti-CCP+	Anti-CCP-	*P*-value
		(*n* = 20)	(*n* = 80)	
Cit-Fil_307 − 324_	14	12 (60.0%)	2 (2.5%)	< 0.01
Arg-Fil_307 − 324_	2	1 (5.0%)	1 (1.2%)	0.47
Cit-Fibα_36−50_	12	10 (50.0%)	2 (2.5%)	< 0.01
Arg-Fibα_36−50_	4	0 (0.0%)	4 (5.0%)	0.70
Cit-Fibα_563−583_	8	7 (35.0%)	1 (1.2%)	< 0.01
Arg-Fibα_563−583_	1	1 (5.0%)	0 (0.0%)	0.27
Cit-Fibα_580−600_	4	4 (20.0%)	0 (0.0%)	< 0.01
Arg-Fibα_580−600_	4	1 (5.0%)	3 (3.8%)	1.00
Cit-Fibα_621−635_	10	9 (45.0%)	1 (1.2%)	< 0.01
Arg-Fibα_621−635_	0	0 (0.0%)	0 (0.0%)	1.00
Cit-Fibβ_36−52_	9	8 (40.0%)	1 (1.2%)	< 0.01
Arg-Fibβ_36−52_	0	0 (0.0%)	0 (0.0%)	1.00
Cit-Fibβ_60−74_	12	12 (60.0%)	0 (0.0%)	< 0.01
Arg-Fibβ_60−74_	3	1 (5.0%)	2 (2.5%)	0.61
Cit-Vim_2 − 17_	3	3 (15.0%)	0 (0.0%)	0.01
Arg-Vim_2 − 17_	1	1 (5.0%)	0 (0.0%)	0.27
Cit-Vim_60 − 75_	17	12 (60.0%)	5 (6.2%)	< 0.01
Arg-Vim_60 − 75_	1	0 (0.0%)	1 (1.2%)	1.00
CEP-1	15	11 (55.0%)	4 (5.0%)	< 0.01
REP-1	1	0 (0.0%)	1 (1.2%)	1.00
Cit-Pept Z1	7	7 (35.0%)	0 (0.0%)	< 0.01
Arg-Pept Z1	2	2 (10.0%)	0 (0.0%)	0.06
Cit-Pept Z2	9	8 (40.0%)	1 (1.2%)	< 0.01
Arg-Pept Z2	5	3 (15.0%)	2 (2.5%)	0.08
Cit-Pept 1	3	3 (15.0%)	0 (0.0%)	0.01
Arg-Pept 1	3	2 (10.0%)	1 (1.2%)	0.15
Cit-Pept 5	14	11 (55.0%)	3 (3.8%)	< 0.01
Arg-Pept 5	3	3 (15.0%)	0 (0.0%)	0.01
Cit-Pept Bla26	10	7 (35.0%)	3 (3.8%)	< 0.01
Arg-Pept Bla26	7	5 (25.0%)	2 (2.5%)	< 0.01

Number of detected antibodies reactivities to the included peptides in the whole JIA cohort (all JIA) and in the anti-CCP–positive and anti-CCP–negative JIA subgroups. %-values in brackets denote the frequency of ACPA-positive individuals within the analyzed patient group. P-values show the comparison of peptide frequencies between anti-CCP–positive and anti-CCP–negative JIA patients. P is adjusted

### Associations between ACPA reactivities and HLA-DRB1 SE alleles in JIA patients

To further explore the association between HLA-DRB1 SE alleles and the presence of specific ACPA reactivities, a detailed analysis was conducted on a subset of 94 patients for whom SE allele data were available (Table 3).

A statistically significant association was observed between reactivity to Cit-Vim_60 − 75_ and the presence of HLA-DRB1 SE alleles, with an odds ratio (OR) of 8.6 (*p* < 0.01; 95% CI: 1.82–40.69). A borderline significant association was noted for Cit-Fibα36–50 (OR = 5.33; *p* = 0.05; 95% CI: 1.08–26.18). However, after adjusting for multiple comparisons, only the association of Cit-Vim_60 − 75_ remained statistically significant at the 0.05 threshold.

**Table 3:** Associations between HLA-DRB1 *SE* alleles and ACPA reactivities in JIA patients

ACPA Reactivity	SE+ (*n* = 47)	SE- (*n* = 47)	OR	95% CI	*P*-value	Adjusted *P*-value (FDR)
Cit-Fil_307 − 324_	8	4	2.21	0.62–7.90	0.35	0.54
Cit-Fibα_36−50_	9	2	5.33	1.08–26.18	0.05	0.29
Cit-Fibα_563−583_	4	2	2.09	0.36–12.02	0.68	0.78
Cit-Fibα_580−600_	3	1	3.14	0.31–31.30	0.62	0.77
Cit-Fibα_621−635_	6	2	3.29	0.63–17.24	0.27	0.50
Cit-Fibβ_36−52_	5	2	2.68	0.49–14.56	0.43	0.59
Cit-Fibβ_60−74_	8	2	4.62	0.92–23.04	0.09	0.30
Cit-Vim_2 − 17_	3	0	∞	—	0.24	0.50
Cit-Vim_60 − 75_	13	2	8.6	1.82–40.69	< 0.01	0.05
CEP-1	7	6	1.2	0.37–3.87	1.00	1.00
Cit-Pept Z1	4	1	4.28	0.46–39.81	0.36	0.54
Cit-Pept Z2	6	1	6.73	0.78–58.28	0.11	0.30
Cit-Pept 1	2	1	2.04	0.18–23.35	1.00	1.00
Cit-Pept 5	9	3	3.47	0.88–13.76	0.12	0.30
Cit-Pept Bla26	7	1	8.05	0.95–68.27	0.06	0.29

HLA-DRB1 SE allele data, shown as SE+ (positive) and SE- (negative) in the table, was available for 94 patients

### Correlations between ACPA reactivities

**Fig. 2:**
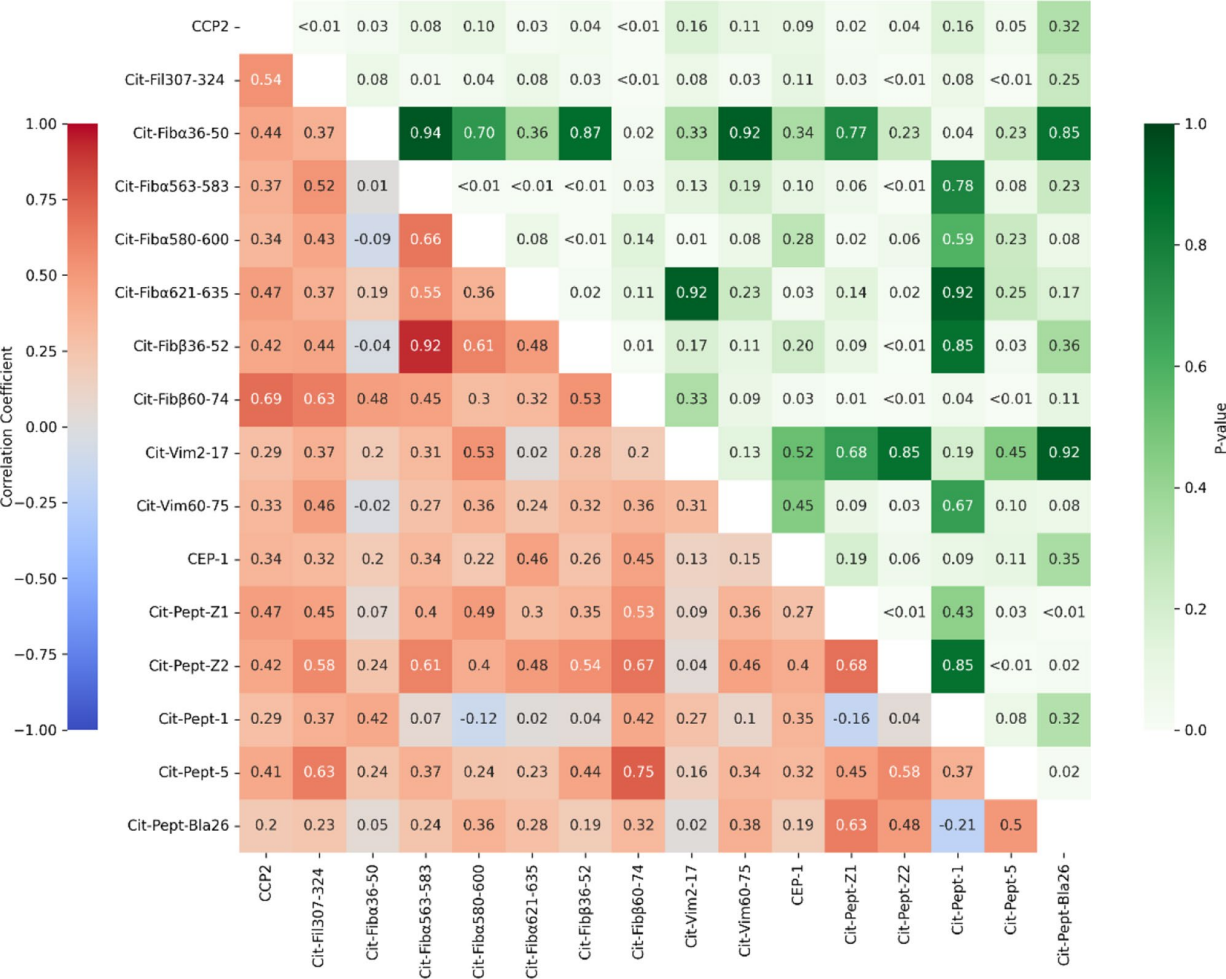
Correlation of citrullinated peptide reactivities. A Spearman correlation matrix of citrullinated peptide reactivities was created. Spearman correlation coefficients are displayed in the lower-left triangle using a coolwarm colour palette. Each cell is annotated with the corresponding correlation value, ranging from − 1 to + 1. FDR-corrected p-values in the upper-right triangle, color-coded using a green gradient. P-values are annotated in each cell, with values below 0.01 displayed as “<0.01” and others rounded to two decimal places. Colour bars indicate the strength of correlation (left) and statistical significance (right). P is adjusted

To investigate the relationships among peptide reactivities, we conducted a Spearman correlation analysis and visualized the results in a heatmap (Fig. 2), where the left triangle displays correlation coefficients, and the right triangle shows corresponding p values. Several strong positive correlations (ρ ≥ 0.5) with statistically significant p values (*p* < 0.01) were observed.

Notably, the strongest correlation was observed between Cit-Fib⍺563–583 and Cit-Fibβ36–52 (ρ = 0.92, *p* < 0.01). Other prominent correlations included Cit-Fibβ60–74 with Cit-Pept-5 (ρ = 0.75, *p* < 0.01) and CCP with Cit-Fibβ60–74 (ρ = 0.69, *p* < 0.01). Additionally, significant correlations were detected between Cit-Pept-Z1 and Cit-Pept-Z2 (ρ = 0.68, *p* < 0.01) and between Cit-Fib⍺563–583 and Cit-Fib⍺580–600 (ρ = 0.66, *p* < 0.01).

## Discussion

Antibodies targeting citrullinated proteins have attracted increasing interest during the last two decades in the field of arthritis due to their high prevalence in RA patients, their ability to establish a diagnosis and evaluate patient prognosis, and their connection to disease pathogenesis [30–34]. ACPAs can be detected years before the clinical onset of RA and have been shown to have multiple biological effects related to disease development [7, 35, 36]. While it might be tempting to generalize this knowledge, the reality is that our understanding of the spectrum of ACPAs in JIA and their overall influence on disease development remains limited. Although there have been reports indicating a positive correlation between ACPA levels and radiological joint damage, as well as the severity of the disease course in children with JIA, the full scope of their impact and the mechanisms involved still require further investigation [14].

It has previously been shown in other JIA populations, and we confirmed in our study of Swedish JIA patients that the frequency of ACPAs is much lower in JIA patients than in RA patients. In our cohort, the overall prevalence of anti-CCP antibodies was 6.2%. The prevalence of anti-CCP has previously been reported on a wide scale, ranging from 2% to 24% in JIA patients [14, 22, 26, 37–40]. This variability can be attributed to the heterogeneity among the studied cohorts, including differences in ethnic background, cohort size, age, and the composition of JIA subtypes. Additionally, methodological variations in the detection of anti-CCP antibodies could also contribute to these divergent findings. In agreement with previous reports, most of the patients in our cohort (42.9%) who tested positive for anti-CCP antibodies were classified as RF-positive polyarthritis patients. However, it is important to note that 28.6% of anti-CCP–positive patients were in the oligoarthritis category, with an additional 19% classified as having RF-negative polyarthritis. These anti-CCP–positive subsets represent a relatively small portion of these specific JIA subtypes, comprising only 3.5% and 4.7%, respectively. A somewhat higher prevalence has been reported previously for RF-negative polyarthritis, at 6.0–7.5% [26, 41], and has been associated with a more aggressive and erosive disease prognosis [41]. In our study, two out of the four undifferentiated arthritis patients were anti-CCP–positive. Previously, up to 80% of undifferentiated JIA patients were reported to be anti-CCP–positive [22]. Notably, the two undifferentiated JIA patients in our cohort also tested positive for RF.

HLA-DRB1 SE alleles are known risk factors for ACPA-positive RA [42] and were also linked to JIA in a previous study [22]. There was a significant association between anti-CCP antibodies, as well as RF, and carriage of HLA-DRB1 SE in our study. Hence, the ACPA-positive subset of JIA may represent a juvenile form of adult ACPA-positive RA. However, symptoms develop at a much younger age, and these patients are less likely to have been exposed to firsthand smoke, which is the best-known environmental risk factor for ACPA-positive RA, particularly in individuals carrying the shared epitope. This highlights the ACPA-positive JIA subset as a unique group of patients for further detailed studies of the etiopathology underlying the development of ACPAs.

An earlier study demonstrated that the prevalence and levels of anti-CCP antibodies were greater in older polyarthritis patients (>16 years old) than in younger patients (< 16 years old) [43]. Similarly, in our study, we observed that anti-CCP–positive JIA patients were older at onset. We did not observe a longer disease duration than in anti-CCP–negative patients, which has previously been reported in JIA [14]. Furthermore, we did not observe that disease duration had a significant effect on anti-CCP levels or the diversity and number of ACPA reactivities (data not shown).

This study highlights distinct ACPA reactivity profiles between anti-CCP–positive and anti-CCP–negative JIA patients. Anti-CCP–positive patients demonstrated broad reactivity, with a marked preference for citrullinated epitopes over their native arginine counterparts. The absence of significant reactivity to native arginine-containing peptides in both groups confirms the citrulline-specific nature of the ACPA response and suggests that citrullination might play a role in the autoimmune response in a subset of JIA.

Strong reactivity to multiple citrullinated epitopes, such as Cit-Fil307–324, Cit-Vim60–75, CEP-1, Cit-Fibβ60–74, and Cit-Fibα36–50, with reactivity rates ranging from 50% to 60% in the anti-CCP–positive group, was observed in our study. This pattern closely resembles the ACPA profile observed in adults with RA, indicating potential shared immunopathogenic mechanisms. These ACPA fine specificities have previously been reported as among the most prevalent in RA, typically at higher frequencies ranging from 60% to 80% in anti-CCP–positive RA patients and 3% to 9% in anti-CCP–negative RA patients [44]. The prevalence of anti-citrullinated vimentin antibodies in JIA has been previously reported to be 5%-9% [25, 39, 45], that of anti-citrullinated fibrinogen has been reported to be 32%, and that of anti-citrullinated α-enolase has been reported to be 9% [24].

In contrast, the anti-CCP–negative group exhibited significantly lower reactivities. This lack of reactivity suggests that anti-CCP–negative JIA may involve different immunological pathways and mechanisms of joint inflammation than anti-CCP–positive JIA, which does not involve citrullination or ACPA-mediated autoimmunity.

Notably, Fibβ_60−74_ exhibited unique characteristics in our study, with 60% reactivity in the anti-CCP–positive group and 0% reactivity in the anti-CCP–negative group and low reactivity of 5% and 2.5%, respectively, in both groups to the Arg peptide. Cit-Fibβ_60−74_ also had a significant correlation coefficient (ρ) of 0.69 with anti-CCP. The strong association between anti-CCP and anti-Cit-Fibβ_60−74_ aligns with earlier reports in RA and indicates that this antibody effectively captures a significant portion of the CCP signal measured by anti-CCP2 ELISA [21, 46].

In our multiplex array analysis, none of the peptides could encompass the entirety of patients who tested positive with the CCP2 ELISA. Conversely, although the majority of ACPA-positive patients were also anti-CCP–positive, 18.7% were in the anti-CCP–negative subset. These data are in line with previous observations in RA, where ACPAs were detected in 16% of seronegative patients, who were classified as anti-CCP- and RF-negative. Interestingly, this group of RA patients resembled seropositive RA patients in terms of their association with HLA-DRB1 SE, higher disease activity over time and worse clinical outcomes [44, 47].

In our study, we found that several ACPA reactivities cooccurred at high frequency, whereas some ACPAs did not. This observation is also in accordance with previous reports in RA [15, 21] and could be explained by different degrees of cross-reactivity, as well as epitope spreading, with an ongoing process of affinity maturation and somatic hypermutation within ACPA-encoding B-cell lineages [48]. Importantly, HLA-DRB1 SE alleles were significantly associated only with the presence of anti-CCP and anti-Cit-Vim_60 − 75_ antibodies. This finding may be attributed to the limited number of observations for each ACPA’s fine specificity, which affects the power of the calculations. Additionally, in RA, SE has been shown to be associated with certain ACPA fine specificities but not others [19]. It has been suggested that ACPA reactivities may have a different genetic architecture, explaining the differential patterns of co-occurrence observed between specific ACPAs and the genetic factors associated with them. This concept aligns with previous reports indicating variations in genetic architecture related to specific ACPA reactivities [21].

This study has several limitations. ACPA fine specificities were assessed with a multiplex array in only a subset of anti-CCP–negative JIA patients. Consequently, the overall prevalence of ACPAs in the cohort may be biased toward anti-CCP–positive patients, and the findings should not be extrapolated to the entire JIA population. Therefore, our analysis of ACPAs focused on comparisons between anti-CCP–positive and anti-CCP–negative patients. ACPAs have previously been linked to a more severe and erosive form of the disease, but no radiographic or disease activity data are available to correlate ACPAs with clinical outcomes. Furthermore, longitudinal studies with new or early-onset JIA will be necessary to further characterize the role of ACPAs in JIA disease prognosis and joint damage.

## Conclusions

In conclusion, our study demonstrated the presence of multiple ACPA reactivities in a Swedish JIA cohort, highlighting the heterogeneity of the ACPA response. These reactivities span several peptide clusters and offer insight into the underlying structure of the autoantibody response in this patient group. Anti-CCP–positive JIA may represent a biologically distinct subset with closer immunopathological ties to ACPA-positive RA. These findings highlight ACPA-positive JIA as a distinct subgroup, offering a valuable model for studying the early molecular events driving the immune response to citrullinated proteins and the development of arthritis. Stratifying JIA patients based on ACPA profiles may have important implications for diagnosis and targeted therapy, warranting further investigation.

## Supplementary Information

Below is the link to the electronic supplementary material.

Supplementary Material 1

## Data Availability

According to Swedish law, individuals’ data are not publicly available, but the datasets generated and/or analysed during the current study are available upon reasonable request.

## Abbreviations

ACPA: Anti-citrullinated protein antibodies
ANA: Anti-nuclear antibodies
CCP: Cyclic citrullinated peptide
FEIA: Fluorescent Enzyme Immunoassay
HLA: Human leukocyte antigen
hnRNP: Heterogeneous nuclear ribonucleoprotein
ILAR: International League of Associations for Rheumatology
JIA: Juvenile idiopathic arthritis
RA: Rheumatoid arthritis
RF: Rheumatoid factor
SE: Shared epitope
