# Supplementary material for: Presence and reactivities of antibodies directed to citrullinated peptides in a Swedish JIA cohort
Source: Pediatr Rheumatol Online J. 2025 Nov 22;23:129. doi: 10.1186/s12969-025-01177-1 (PMC12750833; doi:10.1186/s12969-025-01177-1)
Supplement: Supplementary file 1 — Supplementary Material 1 [file 12969_2025_1177_MOESM1_ESM.docx]

| **Peptide** | **Protein** | **Amino acids** | **Sequence** |
| --- | --- | --- | --- |
| Cit-Fil_307-324_ | Filaggrin | 307-324 | HOCHQEST(cit)GRSRGRCGRSGS |
| Cit- Fibα_36-50_ | Fibrinogen α-chain | 36-50 | GP(cit)VVE(cit)HQSACKDS |
| Cit- Fibα_563-583_ | Fibrinogen α-chain | 563-583 | HHPGIAEFPS(cit)GKSSSYSKQF |
| Cit- Fibα_580-600_ | Fibrinogen α-chain | 580-600 | SKQFTSSTSYN(cit)GDSTFESKS |
| Cit- Fibα_621-635_ | Fibrinogen α-chain | 621-635 | (cit)GHAKS(cit)PV(cit)GIHTS |
| Cit- Fibβ_36-52_ | Fibrinogen β-chain | 36-52 | NEEGFFSA(cit)GHRPLDKK |
| Cit-Fibβ_60-74_ | Fibrinogen β-chain | 60-74 | (cit)PAPPPISGGGY(cit)A(cit) |
| Cit-Vim2-17 | Vimentin | 2-17 | ST(cit)SVSSSSY(cit)(cit)MFGG |
| Cit-Vim60-75 | Vimentin | 60-75 | VYAT(cit)SSAV(cit)L(cit)SSVP |
| CEP-1 | α-enolase | 5-21 | C-KIHA(cit)EIFDS(cit)GNPTVE-C |
| Cit-Pept Z1 | hnRNP | (proprietary) | (proprietary) |
| Cit-Pept Z2 | hnRNP | (proprietary) | (proprietary) |
| Cit-Pept-1 | hnRNP | (proprietary) | (proprietary) |
| Cit-Pept 5 | hnRNP | (proprietary) | (proprietary) |
| Cit-Pept Bla-26 | hnRNP | (proprietary) | (proprietary) |

The arginine-containing peptides have identical amino acid sequences except that they contain arginine residues instead of citrulline. Cit = citrulline.
